# Comparison of Meshing Strategies in THR Finite Element Modelling

**DOI:** 10.3390/ma12142332

**Published:** 2019-07-23

**Authors:** Alessandro Ruggiero, Roberto D’Amato, Saverio Affatato

**Affiliations:** 1 Department of Industrial Engineering, University of Salerno, Via Giovanni Paolo II, nr. 132, 84084 Fisciano, Italy; 2 Escuela Técnica Superior de Ingeniería y Diseño Industrial, Universidad Politécnica de Madrid, Roda de Valencia, 3, 28012 Madrid, Spain; 3 Laboratorio di Tecnologia Medica, IRCCS Istituto Ortopedico Rizzoli, Via di Barbiano, 1/10, 40136 Bologna, Italy

**Keywords:** hip joint, prosthesis, tribology, contact, FEM, mesh

## Abstract

In biomechanics and orthopedics, finite element modelling allows simulating complex problems, and in the last few years, it has been widely used in many applications, also in the field of biomechanics and biotribology. As is known, one crucial point of FEM (finite element model) is the discretization of the physical domain, and this procedure is called meshing. A well-designed mesh is necessary in order to achieve accurate results with an acceptable computational effort. The aim of this work is to test a finite element model to simulate the dry frictionless contact conditions of a hip joint prosthesis (a femoral head against an acetabular cup) in a soft bearing configuration by comparing the performances of 12 common meshing strategies. In the simulations, total deformation of the internal surface of the cup, contact pressure, and the equivalent von Mises stress are evaluated by using loads and kinematic conditions during a typical gait, obtained from a previous work using a musculoskeletal multibody model. Moreover, accounting for appropriate mesh quality metrics, the results are discussed, underlining the best choice we identified after the large amount of numerical simulations performed.

## 1. Introduction

Finite element (FE) modelling as a research tool has been widely used in last few decades to study many different applications in the field of mechanics. Currently, its applications have expanded to simulations of other phenomena also in biomechanics and biomechanical engineering, especially in complex systems that are not accessible experimentally [1,2]. For example, Brekelmans et al. [3] in their study introduced a new method to analyze the mechanical behavior of skeletal parts by applying the new analysis to a simple 2D model of the human femur. Their results demonstrated that finite element analysis is suitable for the study of complex human parts such as the femur. Shim et al. [4] developed an FEM simulation in order to obtain an efficient and accurate prediction model for the stability of percutaneous fixation of acetabular fractures. Their FE model showed that this tool is of fundamental importance for the analysis of different fracture fixation techniques. In fact, their model was able to predict the movement of the fragment with a reasonable accuracy. From a tribological point of view, considering the interaction between the femoral head and the acetabular cup in a Total Hip Replacement (THR), Affatato et al. [1] developed an in silico model in order to investigate the radial clearance influence on the acetabular cup contact pressure in hip implants during a gait cycle. The novel model provided the use of a new soft bearing model (hard-on-soft) FEM taking into account the kinematic and dynamic conditions calculated from a musculoskeletal multibody model during the gait of a person. Ruggiero et al. in [5] presented an FEM simulation of a hip joint prosthesis, and the results were presented in terms of the total deformation and contact pressure of the acetabular polyethylene liner in contact with the femoral head of a hard material. Islán et al. [6], in their study, investigated and simulated, with the use of FEM, the behavior of the glenohumeral joint and the rotator cuff of a musician during his workout in repetitive routines, while in [7], the authors used a FEM approach to investigate the loaded behavior of a human shoulder by including also the ligaments (glenohumeral and coracohumeral) and the glenohumeral capsule in the anatomical model. The authors, in their study, obtained an improvement in the meshing process of the 3D model of human articulation by using a tetrahedron with 10 nodes as the mesh elements. In [6,7], it was also underlined that an important feature of FEM is the mesh type, since it represents the discretization of the virtual domain necessary to transform the continuous body into a finite number of nodes and elements. Since the meshing strategy directly affects the accuracy of the calculated solution, there are some parameters that need to be respected in order to obtain a realistic model with accurate results by using limited computational effort. The generation of the mesh requires making some decisions such as the shape of the elements and their dimensions. The quality of a mesh is then crucially important, in order to define an acceptable tradeoff between the accuracy of the solution and the computational effort. In fact, too coarse a mesh will result in an inaccurate solution, while thick meshes become impracticable from the computational point of view. For this reason, for a better convergence of the numerical solution, a finer mesh is needed, but this condition will increase the computing resources necessary for the simulation. Raut in his study [8] investigated the impact of mesh quality parameters on elements such as beams, shells, and 3D solids by comparing the performance of linear and quadratic tetrahedral elements and hexahedral elements of the mesh, in various structural problems approached by FEM simulations. The study demonstrated that the results obtained with quadratic tetrahedral elements and hexahedral elements were equivalent in terms of accuracy when the number of nodes was the same for both mesh elements. Generally, hexahedral meshes are assumed to produce more accurate results than those obtained with tetrahedral ones, as demonstrated by Tadepalli et al. [9]. Benzley et al. [10] in their investigation reported that mesh composed of tetrahedral elements compared to hexahedral elements led to greater errors in terms of displacement and stress for static bending. The same occurs for the torsion and dynamic loading, due to the stiffness matrix eigenvalues that result in being greater in the first case. Other authors supported this thesis, such as in [9], in which it was stated that tetrahedral elements should be applied in the FEM only under frictionless conditions or when the conditions of material incompressibility can be relaxed. According to these studies, Burkhart et al. [11] suggested that biological structures, human prostheses or human joints, should be meshed with hexahedral elements [12], especially in dynamic models. FEM requires moderate aspect ratios [13,14] in order to optimize the accuracy and the condition of the problem. In the case of a mesh with tetrahedral elements, Tsukerman and Plaks [15] demonstrated that values of the aspect ratio between one and four are acceptable.

One of the techniques used for the evaluation of mesh quality, during FEM simulations, before the analysis is checking the Jacobian ratio for elements [12]. These provide a measure of distortion from an ideally-shaped element. Moreover, they represent the determinant of the Jacobian matrix that defines the mapping of vertices from the ideally-shaped element to the real element. 

The purpose of this study is to develop a dry frictionless contact FEM of a hip joint prosthesis with 12 common meshing strategies in order to compare the difference in terms of the calculated solution. The variation of the dimension of the mesh elements and the kind of mesh selected pointed out the relation between the results and the mesh quality, assessed through the Aspect Ratio (AR), Jacobian ratio, and mesh skewness.

## 2. Materials and Methods

**Figure 1:**
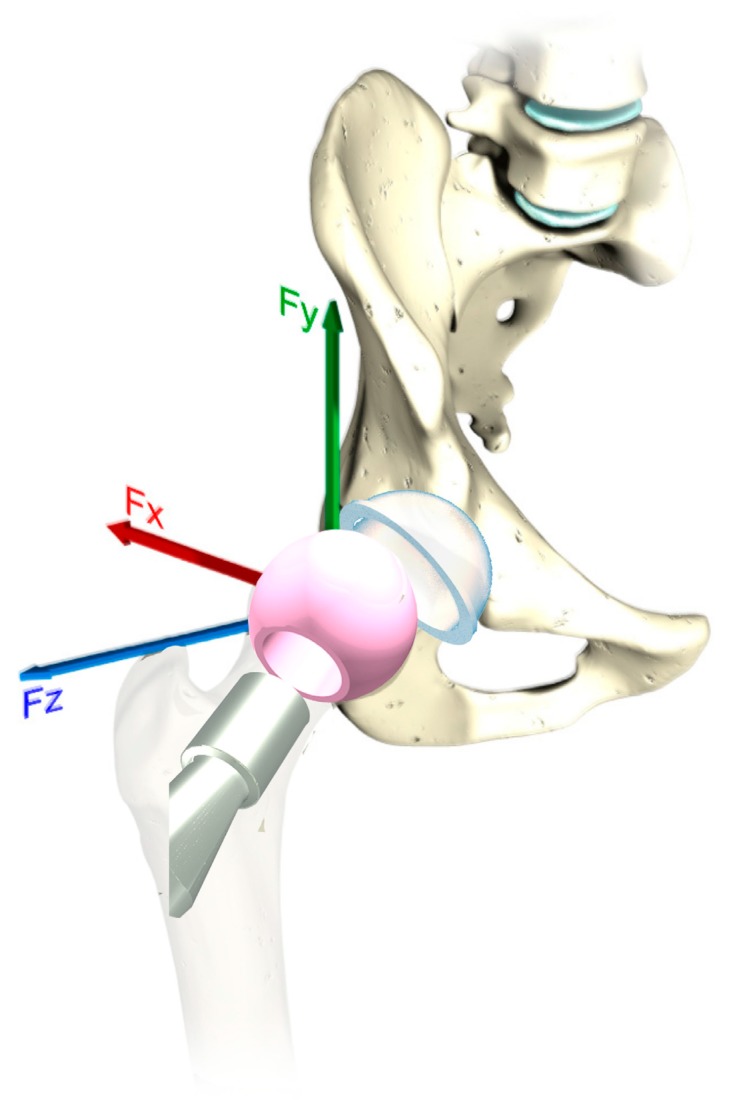
Exploded view drawing of a total hip replacement components: Ultra-High Molecular Weight Polyethylene (UHWMPE) acetabular cup (cyan color component in the picture) and ceramic femoral head (pink component in the picture) with the three force components.

The considered system was the hip joint system used for total hip replacement (Figure 1), focusing our attention on the stress-strain of the femoral head/acetabular cup system during the contact, assuming a frictionless dry condition. The considered system was assumed as a soft bearing (hard-on-soft), characteristic of, for example, the contact between a ceramic femoral head and an Ultra-High Molecular Weight Polyethylene (UHWMPE GUR 1050 material) acetabular cup [16]. 

The head was meshed with a superficial mesh because it was assumed rigid compared to the acetabular cup. For this reason, it was possible to choose only a quadrilateral or triangular mesh for the femoral head, while for the acetabular cup, different meshing algorithms were applied. The meshing was performed by the Ansys^®^ Workbench commercial software (v.18.1, ANSYS Inc., Canonsburg, PA, USA); both tetrahedral elements’ and hexahedral elements’ meshes in several configurations were tested with a total of 12 types of meshes, summarized in Table 1.

The kinematical and dynamical conditions of the systems were selected according to previous works [1,17] in which the prostheses loading was computed considering the result obtained from a musculoskeletal simulation during the gait, splitting up the gait cycle into 500 steps, and executing the simulations for each step, then in correspondence with 500 consecutive load/displacement bearing combinations. Figure 1 shows the coordinate system for the THR used during the FEM analysis for the load condition (F_x_, F_y_, and F_z_) and for the rotations around the axes: Z (flexion/extension movements); X (abduction/adduction movements), and Y (inward/outward movements). The following algorithms were considered:

1) *Automatic meshing method*: An extensible mesh is realized if possible, otherwise a tetrahedral mesh with a *patch-conforming algorithm* is optimized, in which the position of the nodes is performed in an automatic way by the *program-controlled software*.
2) *Tetrahedron/hybrid meshing method*: With this method, it is possible to generate an exclusively tetrahedral mesh. There is the possibility of choosing between the *“patch-conforming”* and *“patch-independent”* algorithm. The first uses *Delaunay triangulation* for tetrahedron formation. The second is based on a spatial subdivision. This algorithm ensures a refinement of the mesh, where necessary, but preserves larger elements where possible, allowing a faster calculation. It is based on the creation of a tetrahedron that incorporates the entire structure, then this is divided up into the required size.
3) *Hex-dominant meshing method*: This generates a completely hexahedral mesh; this option is recommended for inextensible bodies. The elements formed by this type of mesh are smaller than a tetrahedral mesh, and for this reason, it is not recommended for large bodies. The algorithms check that this type of mesh is applicable through the calculation of the normalized area volume ratio; if this is greater than two, it is necessary to pay attention.
4) *Sweep meshing method*: This forces a diffuse mesh on extensible bodies, including axially-extensible bodies. A mesh of this type is preferable on extensible bodies or if it is necessary to calculate the mesh of a body that rotates around an axis.
5) “*Multizone*”: This is a technique that uses an algorithm of the “patch-independent” type, providing the automatic decomposition of the geometry into extensible and free regions. It will achieve a purely hexahedral mesh in structured regions and a free one in unstructured regions. It is possible to choose the shape of the element to use (hexahedral form, hexahedrons, and prisms). If a body has parts that should be discretized through the “multizone” approach and others do not, the former will be discretized according to this option, while the others according to the default method.
6) *Hexa-core:* This will use a bottom-up meshing approach. It will retain the tri-surface or prism mesh, delete the existing tetra-mesh, and remesh the internal volume with a Cartesian approach.

**Figure 2:**
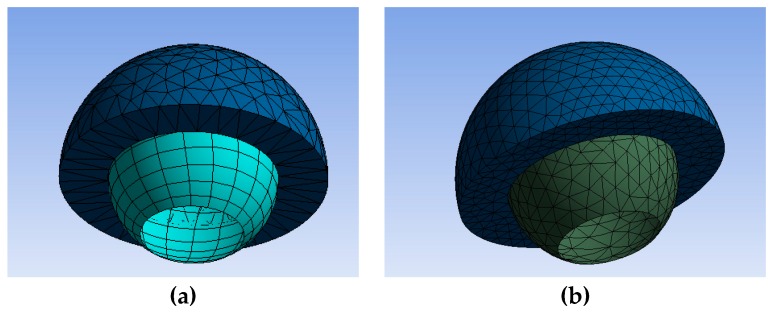
Example of two types of femoral head meshing: (**a**) quadratic tetrahedral and (**b**) triangular tetrahedral.

In Figure 2 is shown an example of two types of femoral head meshing.

Regarding the contact algorithm, the simulations were conducted by using the augmented Lagrange model [5].

For evaluating the global performances of the selected meshing methods, a first investigation was conducted in terms of the numbers of node and numbers of elements. After that, focusing on the meshes with the lowest number of nodes and elements, the Jacobian ratio criteria and the mesh skewness were used. The first one is the ratio between the maximum and the minimum determinant of the Jacobian matrix in different points of the elements [18]. The sampling positions were different according to the kind of elements. In an ideally-shaped element, the determinant should be constant and not change in sign. The value of this metrics should be between 1 and 10, but it is still acceptable to be as high as 30. If the maximum and minimum have different signs, the element is unacceptable. The skewness represents one of the primary quality measures for a mesh since it determines how close to ideal a face or cell is (its value should be as close as possible to zero). The results of the FEM simulation are presented and evaluated on the polyethylene acetabular cup in terms of total deformations, contact pressure, and equivalent von Mises stress.

## 3. Results and Discussion

**Figure 3:**
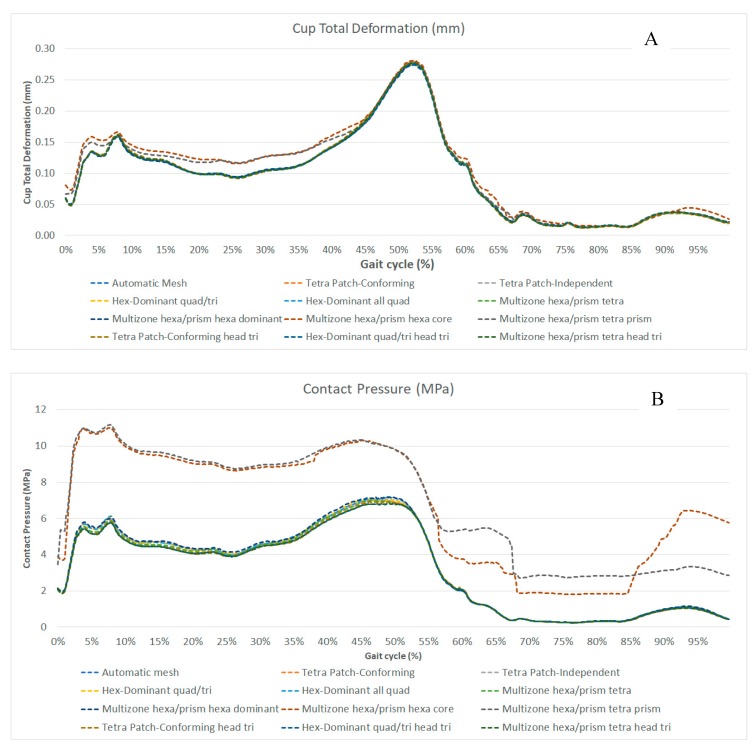

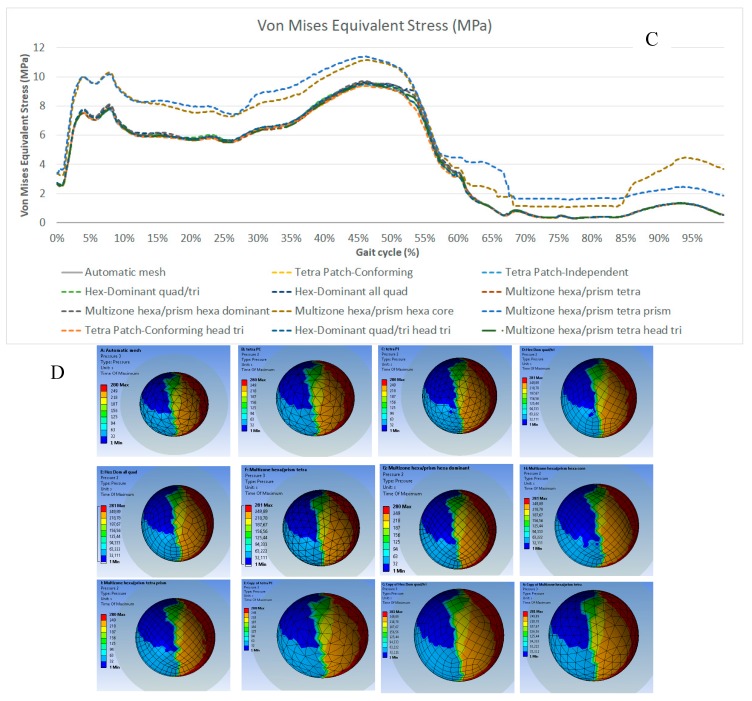
Results of the mesh comparison in terms of: (**A**) the total deformation of the inner cup surface; (**B**) contact pressure; (**C**) von Mises equivalent stress; (**D**) maximum contact pressure time during the gait and the resultant mesh for all meshing algorithms according to Table 1.

In this section, the results of the large number of FE simulations performed are shown by comparing the 12 types of meshes. In particular, first we investigated if the different meshes generated different results in terms of acetabular cup deformation and contact head/cup pressure during the gait cycle. In Figure 3A, the total cup deformations are represented, in Figure 3B, the nodal contact pressure, in Figure 3C, the equivalent von Mises stress, during the gait cycle and for the different mesh types, and in the Figure 3D, the position of the maximum values of the pressure during the walk for all meshing algorithms according to Table 1.

By analyzing Figure 3, it is possible to observe that the results obtained with the different meshes were quite similar, except for two methods: *multizone hexa/prism hexa core* (Mesh 8 in Table 1) and *multizone hexa/prism tetra prism* (Mesh 9 in Table 1), both with an all quad mesh for the femoral head, especially from about 60% of the gait cycle. The triangular mesh for the femoral head was tested only with some of the algorithms compared due to the similarities among the results.

**Figure 4:**
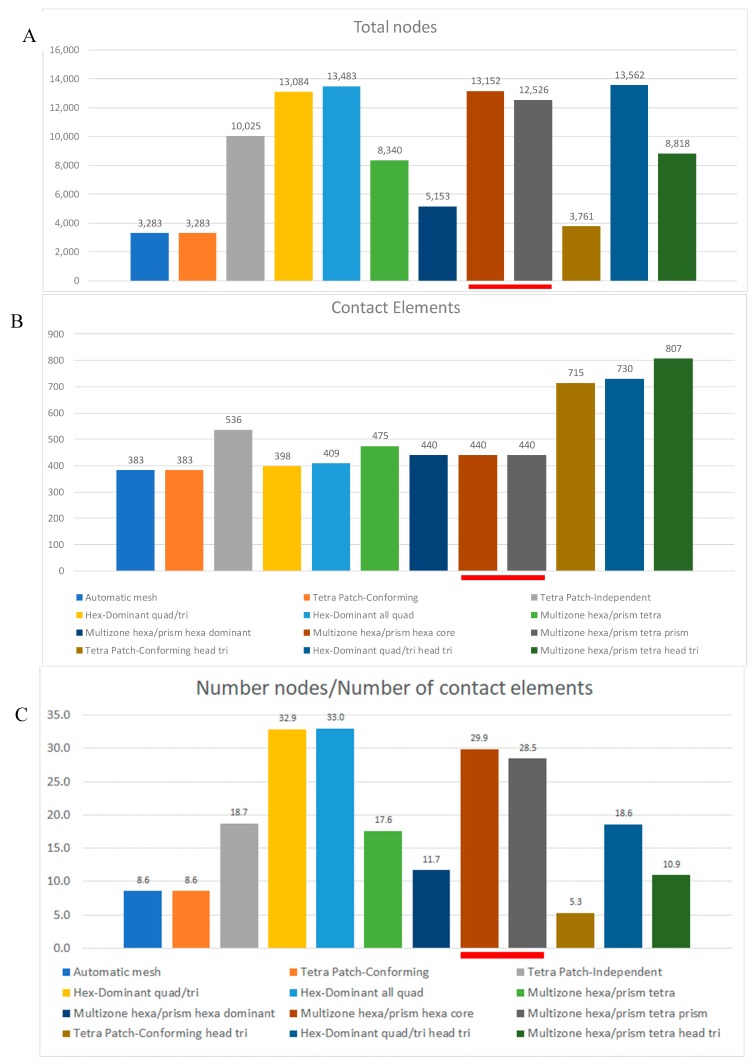
Comparison of the meshes in terms of: (**A**) total number of nodes and (**B**) contact elements; and (**C**) the Coefficient of the Mesh (CM).

Subsequently, the meshes were compared in terms of the number of nodes (Figure 4A), contact elements (Figure 4B), and the Coefficient of the Mesh (CM) evaluated as the ratio between the total number of nodes and the total number of contact elements (Figure 4C) in order to point out the different amounts of computational resources needed.

The three red underlined bars in Figure 4 refer to the two meshes (*multizone hexa/prism hexa core* (Mesh 8 in Table 1) and *multizone hexa/prism tetra prism* (Mesh 9 in Table 1)) with quite different results in terms of contact pressure (Figure 3B) and stress-strain (Figure 3C), which require a deep investigation.

From the analysis of Figure 4B, in terms of contact elements, a larger number of elements, in the case of triangular meshes (Meshes 10, 11, and 12 in Table 1) of the head were found, even if, in dynamical conditions, it is accepted that a quad mesh allows more accurate results than triangular meshes.

**Figure 5:**
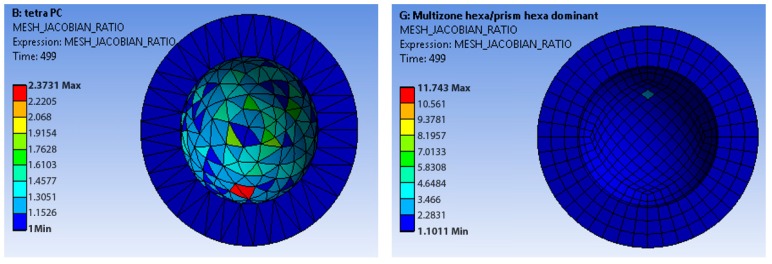
Jacobian ratio of the tetrahedral mesh (**left**) and hexahedral mesh (**right**).

**Figure 6:**
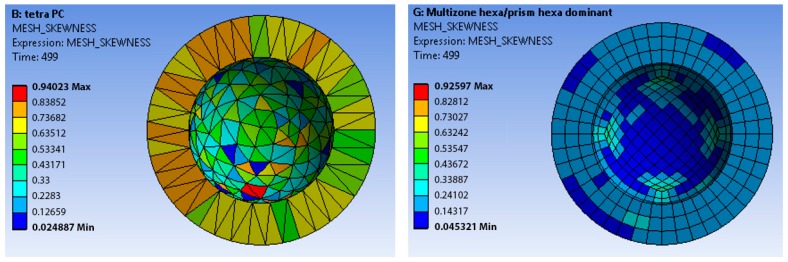
Skewness of the tetrahedral mesh (**left**) and hexahedral mesh (**right**).

Regarding the acetabular cup, Figure 5; Figure 6 show a comparison between the method *tetra patch conforming* (Mesh 2 in Table 1) and the method *multizone* *hexa/prism hexa dominant* (Mesh 7 in Table 1), which are the two meshes with a lower number of nodes. In the first case, the acetabular cup was meshed by tetrahedrons, in the second one by hexahedrons. The femoral head had a quad mesh in both cases. The hexahedral mesh showed values of the Jacobian ratio (Figure 5) closer to the optimum target values. In terms of Aspect Ratio (AR), while the hexahedral mesh showed better values because the AR of its elements was closer to the unity than the elements of the tetrahedral mesh (Figure 6). A similar behavior of the Jacobian ratio in general for the others meshes was also observed. In addition, it is possible to state that the hexahedral mesh had more uniform elements in terms of these two characteristics.

Regarding the skewness, the *hexahedral mesh* was evaluated as more reliable than the *tetrahedral one* (see Figure 6).

As discussed and shown in Figure 3, the two algorithms gave different results from all the others, the algorithms *multizone hexa/prism hexa core* (Mesh 8 in Table 1) and *multizone hexa/prism tetra prism* (Mesh 9 in Table 1). For this reason, more simulations were performed to understand the influence of the nodes and element numbers on the behavior of the solution. It was possible to increase the number of nodes in order to verify if the origin of the observed differences was attributable to a too coarse mesh. A comparison was made in terms of head/cup contact pressure during the gait cycle, because it showed the highest differences in the results (Figure 3B).

**Figure 7:**
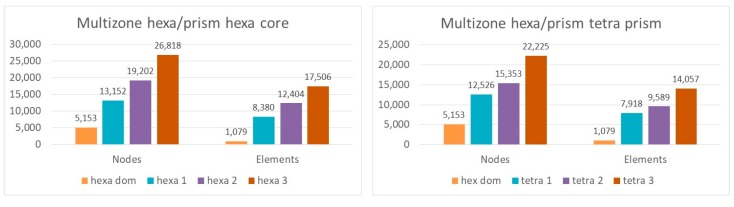
Number of nodes and elements in the different simulations of Mesh 8 and Mesh 9.

The investigated numbers of nodes and elements for both Meshes 8 and 9 are reported in Figure 7.

**Figure 8:**
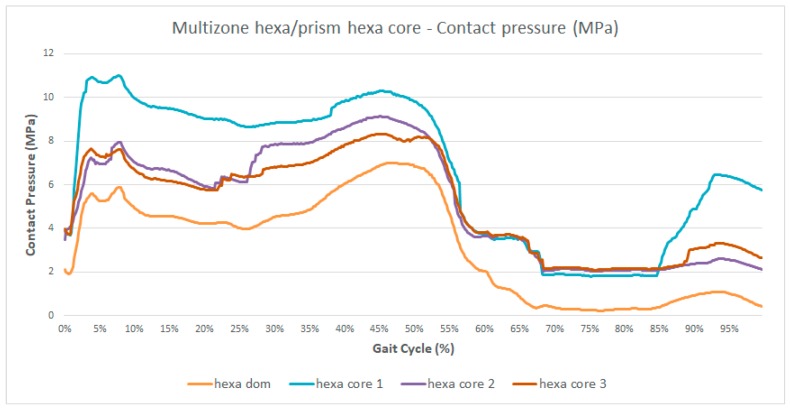
Contact pressure during the gait cycle (%) obtained with the *multizone hexa/prism hexa core* mesh increasing the number of nodes.

**Figure 9:**
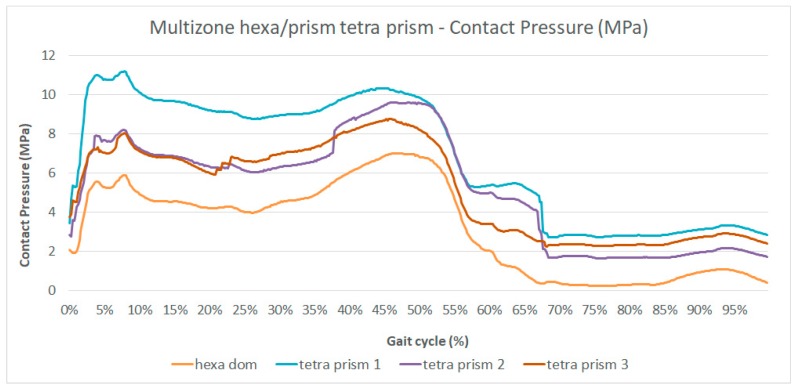
Contact pressure during the gait cycle (%) obtained with the *multizone hexa/prism tetra prism* mesh increasing the number of nodes.

In Figure 8 and Figure 9 are presented the results obtained with *multizone hexa/prism hexa core* (Mesh 8 in Table 1) and with *multizone hexa/prism tetra prism* (Mesh 9 in Table 1) meshes by varying the numbers of elements and nodes in comparison with the *multizone hexa/prism hexa dominant* (Mesh 7 in Table 1) one.

Observing Figure 8 and Figure 9, it is possible to note that both meshes showed a reduction of the differences with respect to the cases in Figure 3, with the increasing number of nodes; the curve shapes and the values of cup stress and deformations during the gait cycle tended to coincide with all other meshes. Therefore, in the framework of this application, the two investigated meshes (Meshes 8 and 9) needed smaller elements, which means grater computational resources in comparison with the other algorithms.

## 4. Conclusions

The in silico approach constitutes a promising methodology in arthroplasty, allowing the prediction of tribological phenomena in preclinical wear tests [19,20,21]. In this study, we performed an in silico approach in order to apply and compare 12 common different meshing strategies on the simulation of the contact between the femoral head and the acetabular cup in a soft bearing. We used the kinematic and dynamic data obtained by a previously-developed musculoskeletal multibody model. The total deformation of the inner surface of the acetabular cup, the head/cup contact pressure, and the equivalent von Mises’ stress were evaluated accounting for the augmented Lagrange contact model. The solution of the simulations showed generally quite similar results except for two meshing algorithms: the *multizone hexa/prism hexa-core* and the *multizone hexa/prism tetra prism*, both with an all quad mesh for the femoral head (the triangular meshing for the femoral head surface showed no relevant differences).

Focusing on the best performing meshes, *tetra patch conforming* (Mesh 2 in Table 1) and the method *multizone hexa dominant* (Mesh 9 in Table 1), the Jacobian ratio criteria and the mesh skewness were computed. The hexahedral mesh showed values of the Jacobian ratio and of the aspect ratio closer to the optimum target values. In terms of the aspect ratio, the hexahedral mesh showed better values. A similar behavior of the Jacobian ratio in general for the others meshes was found, and the hexahedral mesh had more uniform elements in terms of these two characteristics.

Regarding the two meshes, *multizone hexa/prism hexa core* and *multizone hexa/prism tetra prism*, in this particular application, the improvement of the accuracy of the solutions by increasing the number of elements and nodes was observed, concluding that the above algorithms need smaller elements (thick meshes), which means greater computational resources in comparison with the other algorithms.

The comparison between the *tetrahedral mesh* and the *hexahedral mesh* allowed us to conclude the better quality of the latter mesh, especially in dynamic conditions.

## Figures and Tables

**Table 1:** Meshing algorithms.

Cup Mesh	Head Mesh	Mesh Number
Automatic mesh		1
Tetra patch conforming		2
Tetra patch independent		3
Hex dominant quad/tri		4
Hex dominant all quad	All Quad Head	5
Multizone hexa/prism tetra		6
Multizone hexa/prism hexa dominant		7
Multizone hexa/prism hexa core		8
Multizone hexa/prism tetra prism		9
Tetra patch-conforming head tri		10
Hex-dominant quad/tri	Triangular Head	11
Multizone hexa/prism tetra		12
